# Burden of Pneumonia and Meningitis Caused by *Streptococcus pneumoniae* in China among Children under 5 Years of Age: A Systematic Literature Review

**DOI:** 10.1371/journal.pone.0027333

**Published:** 2011-11-16

**Authors:** Ying Chen, Wei Deng, Song-Mei Wang, Qi-Mei Mo, Huan Jia, Qun Wang, Song-Guang Li, Xiang Li, Bao-Dong Yao, Cheng-Jun Liu, Yi-Qiang Zhan, Chen Ji, Anna Lena Lopez, Xuan-Yi Wang

**Affiliations:** 1 Institutes of Biomedical Sciences, Fudan University, Shanghai, People's Republic of China; 2 Department of Health Statistics and Social Medicine, School of Public Health, Fudan University, Shanghai, People's Republic of China; 3 Laboratory of Medical Molecular Biology, Shanghai Medical College, Fudan University, Shanghai, People's Republic of China; 4 Pfizer Inc., New York, New York, United States of America; 5 Key Laboratory of Medical Molecular Virology, Shanghai Medical College, Fudan University, Shanghai, People's Republic of China; Centers for Disease Control & Prevention, United States of America

## Abstract

**Background and Methods:**

To understand the burden and epidemiology of *Streptococcus pneumoniae* disease among children between 1 and 59 months of age in China, we conducted a review of literature published between 1980 and 2008 applying standardized algorithms. Because of the absence of population-based surveillance for pneumococcal disease (PD), we identified all-cause pneumonia, bacteremia and meningitis burden, syndromes most commonly associated with *S. pneumoniae*, and applied the proportion of disease attributable to *S. pneumoniae* from studies that determined the etiology of these three syndromes to calculate PD burden. Because of the microbiologic difficulties in identifying *S. pneumoniae*–attributable pneumonia which likely underestimates the pneumonia burden, we also used the proportion obtained from vaccine efficacy trials.

**Results:**

Between 1980 and 2008, there were 12,815 cases/100,000/year of all-cause pneumonia among children between 1 month and 59 months, with 526 deaths/100,000 annually. There were 14 meningitis cases/100,000/year. We estimate that as of 2000, there were 260,768 (113,000 to 582,382) and 902 (114–4,463) cases of pneumococcal pneumonia and meningitis, respectively with 10,703 (4,638–23,904) and 75 (9–370) pneumococcal pneumonia and meningitis deaths, respectively. Pneumococcal pneumonia cases and deaths were more than two-fold higher, 695,382 (173,845–1,216,918) and 28,542 (7,136–49,949), respectively, when parameters from efficacy trials were used. Serotypes 19F, 19A and 14 were the most common serotypes obtained from pneumonia/meningitis patients. Currently available vaccines are expected to cover 79.5% to 88.4% of the prevalent serotypes. With high antibiotic resistance, introducing pneumococcal vaccines to the routine immunization program should be considered in China. Population-based studies are warranted.

## Introduction

Pneumococcal disease (PD) is the leading cause of vaccine-preventable deaths worldwide [1] accounting for ∼800,000 deaths annually among children <5 years of age [2], [3], [4]. The disease caused by *Streptococcus pneumoniae*, may be invasive resulting in high case-fatality rates of 5% to 20% and 40% to 50% for bacteremia and meningitis, respectively, and in long-term sequelae [5], [6], [7]. PD may also be non-invasive such as pneumonia where majority of the burden lie considering that ∼50% of severe pneumonia is believed to be pneumococcal in etiology (2). However, due to technical difficulties, limitations in access to care and prior antimicrobial use, estimating PD burden remains challenging [8], [9].

Widespread use of pneumococcal conjugate vaccines (PCV) in infants and children resulted in substantial declines in invasive PD (IPD), pneumonia, and otitis media [10], [11], [12], [13], [14]. However, most countries in Asia, including China have not adopted PCVs in their national immunization schedules due to a multitude of reasons including costs, logistic difficulties in introducing a new vaccine and paucity of disease burden data. The limited serotype coverage of PCV7 was also a disincentive in some countries to introduce the vaccine.

Little is known regarding the burden of *S. pneumoniae*-related diseases and the predominant serotypes causing disease in China [15]. Because the distribution of pneumococcal serotypes vary with age, geographic region and time [16], it is crucial to ascertain the circulating pneumococcal serotypes and the corresponding coverage of available pneumococcal conjugate vaccines [17]. This comprehensive systematic review of available English and Chinese literature of studies conducted between 1980 and 2008 aimed to identify the disease burden and serotype distribution of pneumococcal disease in children under 5 years of age in China. A complete understanding of the pneumococcus-associated disease burden in China is critical for developing evidence-based disease burden estimates upon which control measures such as immunization may be based.

## Methods

No population based study determining the burden of pneumococcal disease has been conducted in China, therefore we first, determined the burden of all-cause pneumonia, bacteremia and meningitis, three syndromes most commonly associated with *S. pneumoniae*. Since disease rates may be underestimated when children do not reach the health care facilities, some studies may present incidence rates adjusted for the proportion of missed cases. These adjusted rates were considered whenever they were reported.

Next, from studies that determined the etiology of these three syndromes, we obtained the proportion of disease attributable to *S. pneumoniae*. By applying these proportions to the burden of all-cause pneumonia, bacteremia and meningitis, estimates of pneumococcal pneumonia, bacteremia and meningitis were obtained. For bacteremia and meningitis, culture positive blood or CSF specimens were considered sufficiently sensitive to provide relatively precise estimates of disease burden. However, for pneumonia, the proportion we obtained from microbiologic studies may not be sensitive enough to ascertain the true proportion of pneumonia attributable to *S. pneumoniae* and is more likely an underestimate of the true burden. Thus, we also used the efficacy estimate for WHO-defined clinical pneumonia as previously reported by O'Brien et al. [4]. This proportion was then applied to all-cause pneumonia estimates from this review to obtain pneumococcal pneumonia burden.

### Search strategy

Studies on pneumonia, meningitis and bacteremia caused by *S. pneumoniae*, published between 1980 and 2008, were identified using standardized search algorithms for systematic reviews [18]. Scientific articles published in English and Chinese languages were sought through searching PubMed (United States), Ovid online (United States), SinoMed (Chinese Bio-Medical Literature Service System, China), CNKI (National Knowledge Infrastructure, China) databases. Standardized medical subject heading (MeSH) terms: Meningitis, Bacterial; Pneumonia; Bacteremia; Pneumonia, Pneumococcal; *Streptococcus pneumoniae*, and Free words: China, were established for searching.

Prior to the literature search, a pilot study was conducted to refine the MeSH terms and combinations thereof, especially when the terms were translated to Chinese, prior to searching non-English electronic databases.

### Definitions

Cases of meningitis were defined as having signs of meningeal inflammation with or without isolation of bacterial pathogen. Bacteremia was defined as the presence of pathogenic organisms in the bloodstream. Because of the expected heterogeneity in the definition of pneumonia among the Chinese studies, a more inclusive definition for pneumonia was used to estimate the proportion of all- cause pneumonia episodes that were pneumococcal in etiology. The studies used the following definitions for pneumonia: (1) WHO definition for clinical pneumonia [19], (2) Respiratory Society of the Chinese Medical Association community acquired pneumonia guidelines for diagnosis and treatment (Defined as the presence of a new infiltration on chest radiographs and at least one of the following: documentation of a new cough with or without sputum production or exacerbation of chronic respiratory diseases with purulent sputum production with or without chest pain; documented fever (37.3°C); auscultatory findings on pulmonary examination and/or evidence of pulmonary consolidation; and white blood cells (WBC) count>10×10^9^/L or leucopenia (WBC<4×10^9^/L) [20]. and (3) physicians' assessment with or without radiologic findings.

### Review strategy

Endnote® (version X, Thomson, Inc., Philadelphia, U.S.A.) bibliographic software was used to create an electronic library of citations identified in the database searches. PubMed searches were performed using Endnote® and duplicate records were deleted. For articles published in Chinese and English, if identical data were presented, the English language article was used. Each study was assigned a unique identification code to enable tracking of reviews and analysis after title/abstract screening. Eight reviewers trained to perform the title/abstract screening and thereafter full text screening were assigned into two groups. The inter-reviewer agreements were calculated and kappa values were presented. Appraisal of quality based on the study design, patient recruitment, case definitions and diagnostic methods, was performed for those articles that fulfilled the inclusion criteria using a structured questionnaire. In order to identify the differences in homogeneity of the published articles, each question was assigned a score, with 0 being the lowest and 5 being the highest. Mean points per question were calculated for each article. Articles with ≥3.0 points/question were assigned to moderate/high quality. A structured questionnaire was used for data extraction, and the EpiData (version 3.1) program was used for data entry.

### Study inclusion and exclusion criteria

All studies published from 1980 to 2008 in English and non-English languages, obtained from the sources defined above were assessed.

Studies had to fulfill the following criteria to be included: 1) include children between 1 month and 5 years of age; 2) conducted in mainland China; 3) has extractable data on pneumonia, meningitis and bacteremia; 4) did not exclusively pertain to clinical diagnostic methods, purified basic research, therapies, case reports (single case), health education, policy analysis and case-control studies; 5) conducted after 1980 or disaggregation allows to identify cases after 1980; 6) the surveillance period was at least 12 months 7) were laboratory-based studies on the different etiologic agents with ≤100 pathogens or if the study is on antimicrobial resistance of *S. pneumoniae* had ≤50 isolates

### Analytical strategy and statistical analysis

SAS statistical software was used for analysis (SAS Institute Inc., Cary, North Carolina, USA). PD burden was estimated through multiplying the proportions of pneumonia, meningitis and bacteremia caused by *S. pneumoniae* with the mortality and morbidity of pneumonia, meningitis and bacteremia, using the data summarized from this review, and 2000 national census. For pneumonia, we also applied the proportion obtained by O'Brien et al. [4]. For overall mortality, morbidity, and antimicrobial resistance, the pooled average from all studies and high quality articles (mean score per question ≥3.0) were calculated.

To pool data from available studies, meta-analysis with random-effects model was performed to calculate the point estimate and 95% confidence interval (CI), followed by logarithmic transformation of individual proportion or rate. If zero events occurred, a correction factor of 0.5 was applied. When the number of original studies was less than four, meta-analysis with random-effects model was only conducted to obtain an average estimate; the 95% CI was presented accordingly [21], [22], [23], [24]. To assess differences in geographic regions, poison regression was used.

### Ethics

This study was reviewed and approved by the Institutes of Biomedical Sciences Institutional Review Board, Fudan University.

## Results

### Overview of studies

**Figure 1:**
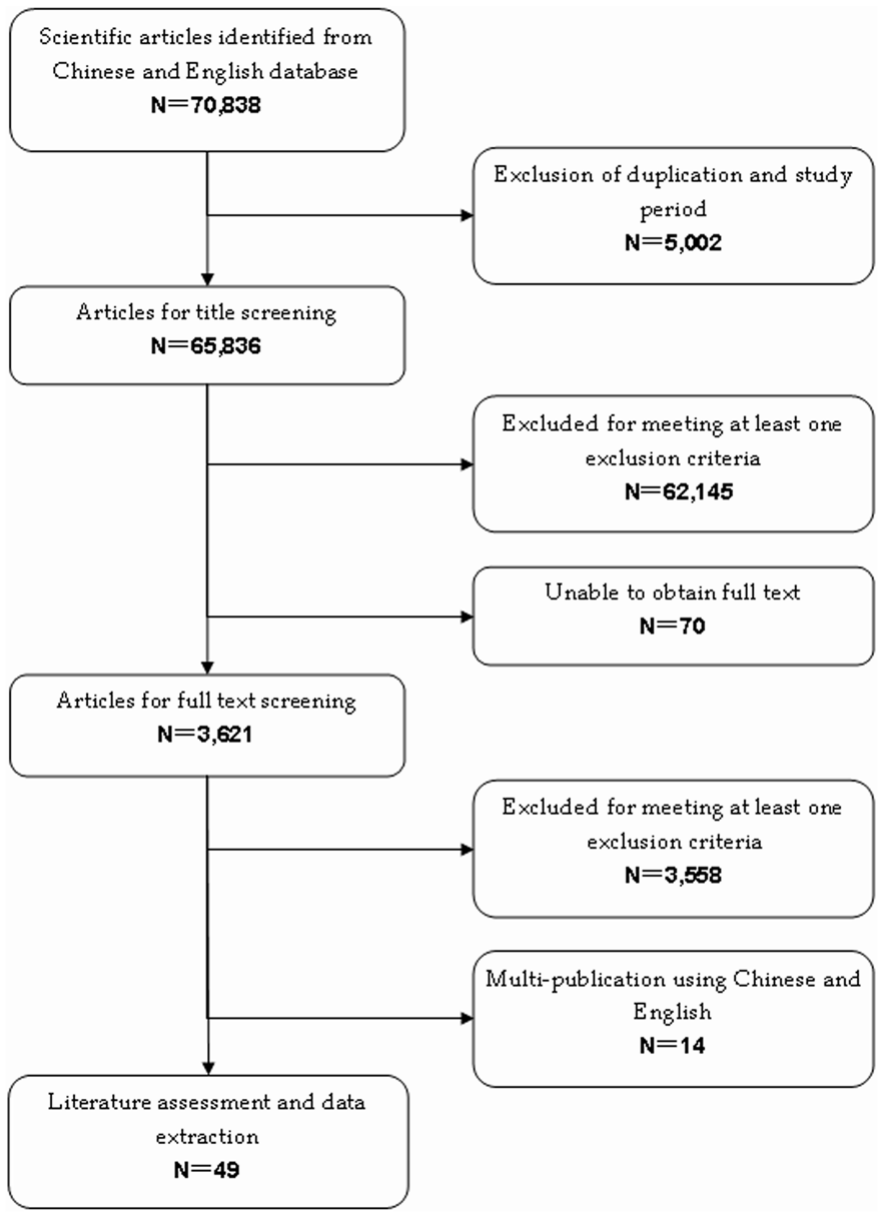
Eligibility of studies for inclusion in systematic review, China.

The initial search identified 70,838 pneumonia-, meningitis- and bacteremia-related citations. After exclusion of duplicate records, 65,836 and 3,621 studies were excluded following review of title/abstract and full text information, respectively (Figure 1). The inter-observer agreement was 85% and 57% (P 0.05) for title/abstract and full text screen, respectively. Forty-nine published studies met the inclusion and exclusion criteria for this review and were included in the final analysis. Of these, only three (6.1%) studies were published in English. After quality assessment, 95.9% (n = 47) studies were labeled with higher mean score (≥3.0/questions). For all-cause pneumonia-, meningitis- and bacteremia-related citations, 42.9% (n = 21) were hospital-based, 30.6% (n = 15) were community-based and 26.5% (n = 13) laboratory-based surveillance. Out of 12 studies reporting on all-cause pneumonia burden, three used the WHO definition of pneumonia, five used the Chinese Medical Association guidelines. and four were based on physicians' diagnoses. Majority of the studies were from the eastern provinces (n = 28, 57.1%), followed by the central provinces (n = 10, 20.4%) and the western provinces (n = 6, 12.2%). 41 articles presented the results of antimicrobial resistance of which 56.1% (n = 23) reported only results of disk-diffusion tests whereas 43.9% (n = 18) reported the minimum inhibitory concentration (MIC). Data on *S. pneumoniae* bacteremia unrelated to pneumonia or to meningitis were not available.

### Morbidity, mortality and CFR of all-cause pneumonia and meningitis

**Figure 2:**
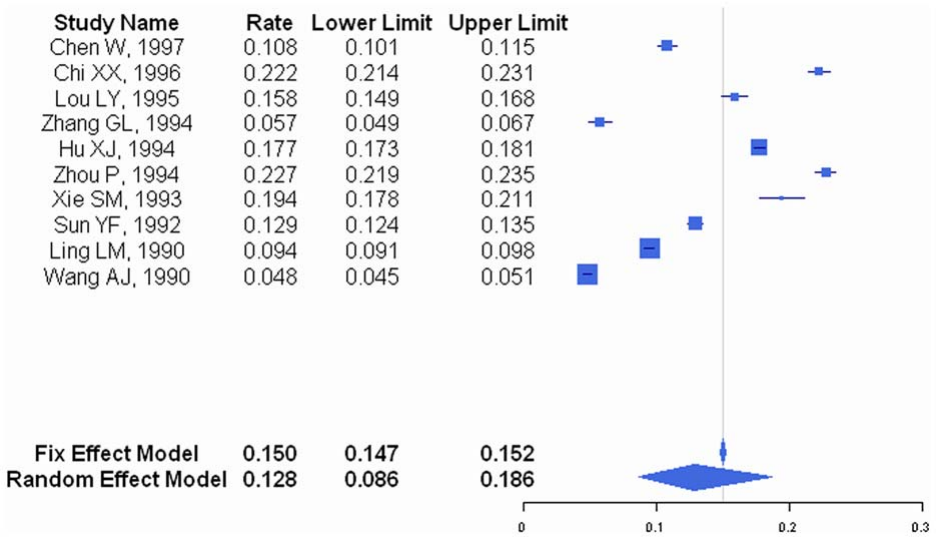
Forest plot of pneumonia morbidity.

**Figure 3:**
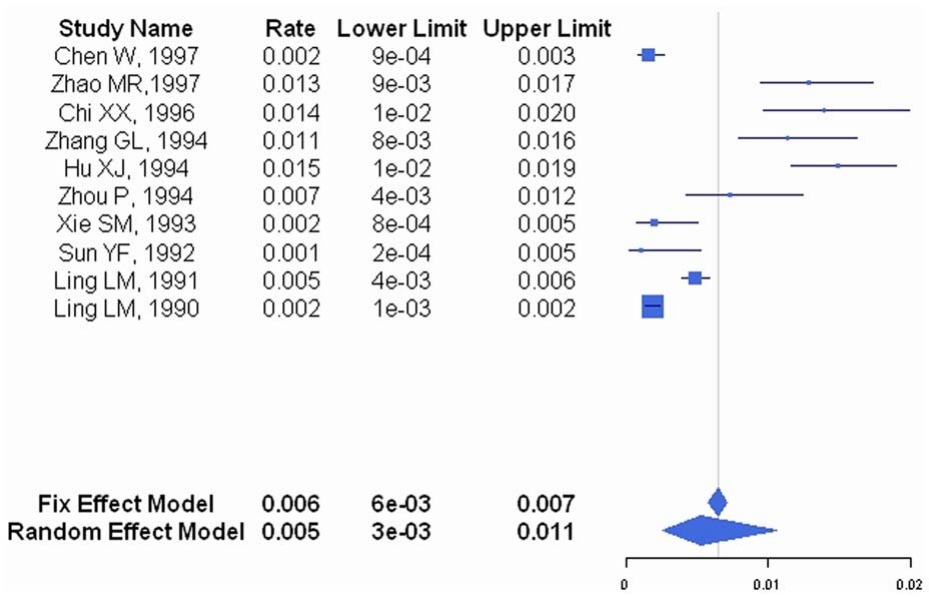
Forest plot of pneumonia mortality.

Between 1980 and 2008, the occurrence of all-cause pneumonia among children aged between 1 month and 59 months was 12,815/100,000/year (95% CI: 8625–18623/100,000/year) with a mortality rate of 526/100,000/year (95% CI: 262/100,000/year–1053/100,000/year) (Table 1, Figure 2, Figure 3). Among the 12 papers that reported on pneumonia incidence, 10 used a clinical definition of pneumonia and two papers used radiologic and/or clinical diagnosis. The addition of the two papers did not alter the results of pneumonia incidence (data not shown).

**Table 1:** Mortality, morbidity, and case-fatality rate due to pneumonia, meningitis and bacteremia, 1980–2008^a^^,^ b ^,^ c.

Disease	Morbidity		Mortality		Case-fatality	
	Rate (n^d^)	95%CI	Rate (n)	95%CI	Rate (n)	95%CI
Pneumonia by year (n = 12)						
1980–1989	6935.6(3)	4775.6, 9444.9, 7305.9^e^	520.2(3)	187.4, 479.9, 1565.6^e^	1.4(2)	2.0, 1.0^e^
1990–1999	14210.9(8)	8663.1–22438.6	551.4(8)	237.8–1273.7	1.3(2)	1.4,0.9^e^
Overall	12815(10)	8625–18623	526(10)	262–1053	1.4(4)	0.7–2.5
Pneumonia by region (n = 10)						
Eastern provinces	18807.0(3)	22203.2,12909.1,22701.8^e^	577.9(3)	1364.7, 71.2, 700.8	-	-
Central provinces	8370.6(4)^f^	3323.2–19535.2	623.8(3)	1263.8, 151.6, 1158.1	1.4(1)	-
Western provinces	18347.9(2)	19377.8, 17700.1^e^	574.9(2)	178.1, 1469.9	0.9(1)	-
Meningitis (n = 4)						
Overall	14(2)	8.9, 19.2^e^	-	-	8.3(3)	18.4, 9.1, 2.5^e^

a Mortality and morbidity were expressed as 1/100,000/year; case-fatality rate was expressed as percentages.

b The mean score per question of all articles was ≥3.0.

c the Ministry of Health-defined geographic-economic strata, which was applied in the 4th national health survey in 2005 [47] was implemented.

d n represents the number of articles.

e Numbers presented are from the original publication.

f Compared to Eastern and Western province, poisson regression, p<0.05.

There were 14 meningitis cases/100,000/year (95% CI: 0.1-1579.4/100,000/year). Regardless of the study year, similar pneumonia morbidity rates were found in eastern and western provinces, and lowest in central provinces (p = 0.009), while mortality rates in the three regions were not significantly different.

### Proportion of S. pneumoniae infections and nasopharyngeal carriage of S. pneumoniae among ill children <5 years of age

Out of five studies with 971 CSF culture specimens from meningitis cases, 142 *S. pneumoniae* were identified (pooled percentage 9.5%, 95% CI 1.2–47.1). Nine studies reported 9,579 specimens from tracheal aspirates, bronchoalveolar lavage, pleural fluid and lung puncture from patients presenting to the hospital with pneumonia, among which 502 specimens yielded *S. pneumoniae* (pooled percentage 3%, 95% CI, 1.3–6.7). Two studies with 1,455 nasopharyngeal swabs from patients presenting with pneumonia or meningitis had 387 *S. pneumoniae* identified (pooled percentage 27%, 95% CI 25.8–29.2).

### Distribution of serotypes from ill children <5 years of age

Only one out of four papers that reported on serotype distribution presented results from invasive specimens separately. The most common serotypes among invasive isolates were 19A (29%), 19F (22.5%) and 14 (12.9%). These serotypes were also among the top five most commonly identified serotypes from mixed specimens, namely: 19F (35.7–70.9%), 19A (9.7–11.8%), 23F (5.8–24.1%), 6B (5.1–7.3%) and 14 (2.9–7.8%) (Table 2). Our findings show that the putative coverage of the 7–10- and 13-valent pneumococcal conjugate vaccines were 79.5% (95%CI: 47.9–94.3%), 79.7% (95%CI: 48.5–94.2%) and 87.2% (95%CI: 65.0–96.1%), respectively of isolates from mixed specimens.

**Table 2:** Serotype distribution of *S. pneumoniae* isolates from patients with pneumonia, meningitis and bacteremia^a^.

	Studies (Publication Year)									
	Zhao GM, et al. 2003		Liu Y, et al.2008				Deng Q, et al. 2008		Yao KH, et al.2008	
Specimen type	Sputum		Sputum, purulent discharge		Blood, CSF, Pleural fluid		Sputum		Hypopharyngeal aspirates	
Serotype	n	%	n	%	n	%	n	%	n	%
4	**-**	**-**	**-**	**-**	**-**	**-**	2	2.5	8	2.9
6B	6	5.4	33	7.9	-	-	4	5.1	15	5.4
14	8	7.1	31	7.4	4	12.9	-	-	8	2.9
19F	40	35.7	183	43.5	7	22.5	56	70.9	169	60.6
23F	27	24.1	25	6	1	3.2	13	16.5	26	9.3
5	-	-	-	-	3	9.7	-	-	1	0.4
3	-	-	-	-	-	-	-	-	1	0.4
6A	10	8.9	-	-	-	-	-	-	4	1.4
19A	-	-	44	10.4	9	29	-	-	27	9.7
2	-	-	-	-	-	-	-	-	1	0.4
10A	-	-	-	-	-	-	-	-	1	0.4
11A	-	-	-	-	-	-	-	-	1	0.4
15B	-	-	-	-	-	-	2	2.5	3	1.1
33F	-	-	-	-	-	-	-	-	1	0.4
11	-	-	2	0.5	2	6.5	-	-	-	-
15	-	-	20	4.8	-	-	-	-	-	-
Non-typable	8	7.1	-	-	-	-	2	2.5	13	4.7
Others	13	11.6	82	19.5	5	16.2	-	-	-	-

a All studies were published between 2000 and 2008, and mean score per question was ≥3.0.

### Profile of antimicrobial resistance

Twenty articles reported on antimicrobial resistance. Of these, three articles included results from invasive isolates but the results cannot be disaggregated and 17 studies (85%) reported on nasopharyngeal swabs from ill patients or sputum specimens. All the articles included in this review used the old Clinical and Laboratory Standards Institute (CLSI) breakpoint for penicillin resistance [25] and reported as such. Regardless of study quality, the articles reported high rates of antimicrobial resistance (Table 3). There was a trend towards increasing antimicrobial resistance for erythromycin (44.2, 95%CI: 0–80.1 vs 84.1, 95% CI: 71.2–91.9) and penicillin (11.5, 95%CI: 0–11.5 vs 16.1, 95%CI: 8.2–29.2) between 1990–1999 and 2000–2008, although these were not statistically significant. One article reported 100% resistance to azithromycin while another article reported susceptibility to vancomycin was 76.6% (95%CI: 0–100).

**Table 3:** Antimicrobial resistance^a^ profile of *S. pneumoniae* isolates from both invasive and non-invasive isolates^b^.

Antibiotics	No. of articles	Specimens tested	Resistance	
			n	Pooled rate (95%CI)
Erythromycin	21	6371	4349	81.7(66.7–90.8)
Penicillin	20	5746	1059	15.6(8.2–27.7)
Cefuroxime	14	4780	700	19.2(9.4–35.1)
Ceftriaxone	14	4628	450	5.0(1.9–12.7)
Ofloxacin	4	738	36	2.7(0.2–25.9)
Amoxicillin	8	1908	67	3.3(0.7–13.7)

a Articles with mean score per question ≥3.0 showed similar results. Articles included in the review used old CLSI breakpoints for Penicillin resistance.

b Data cannot be aggregated separately for invasive isolates and are therefore presented here as mixed with non-invasive isolates.

### Estimates of the burden for S. pneumoniae-specific –pneumonia and -meningitis

Using the 2000 China census data of 67,828,891 children <5 years of age and the parameters obtained in our review, we estimate that there were 260,768 (113,000–582,382) pneumonia and 902 (114–4,463) meningitis cases caused by *S. pneumoniae*. Furthermore, the estimated deaths due to *S. pneumoniae* pneumonia and meningitis were 10,703 (4,638–23,904) and 75 (10–370), respectively. Applying the proportions obtained by O'Brien et al. for pneumonia, we estimate that there were 695,382 (173,845–1,216,918) pneumococcal pneumonia cases and 28,542 (7,136–49,949) pneumococcal pneumonia deaths among children <5 years of age in 2000 in Mainland China. (Table 4).

**Table 4:** Estimates of pneumonia and meningitis disease burden among children <5 years of age caused by *S. pneumoniae* in 2000, China.

Variables	Code	Value
**Parameters**		
2000 census <5 yrs	A	67,828,891
Incidence of pneumonia	B	12815 per 100,000
Mortality of pneumonia	C	526 per 100,000
Incidence of meningitis	D	14 per 100,000
Case-fatality rate of meningitis	E	8.3%
*S. pneumoniae* isolation rate for pneumonia	F	3% (1.3%–6.7%)
*S. pneumoniae* isolation rate for meningitis	G	9.5% (1.2%–47%)
**Estimates based on parameters derived from this review**		
Total cases of pneumonia caused by *S. pneumoniae*	H = B*A*F	260,768 (113,000–582,382)
Total deaths of pneumonia caused by *S. pneumoniae*	I = C*A*F	10,703 (4,638–23,904)
Total cases of meningitis caused by *S. pneumoniae*	J = D*A*G	902 (114–4,463)
Total deaths of meningitis caused by *S. pneumoniae*	K = D*A*E*G	75 (9–370)
**Estimates using pneumonia parameters from O'Brien, et al.**		
*S. pneumoniae isolation rate for pneumonia*	F′	8% (2%–14%)
Total cases of pneumonia caused by *S. pneumoniae*	H = B*A*F′	695,382 (173,845–1,216,918)
Total deaths of pneumonia caused by *S. pneumoniae*	I = C*A*F′	28,542 (7,136–49,949)

## Discussion

Our study confirms that pneumococcal disease is a significant health problem among children <5 years in China. Using the parameters that we obtained in our study, our estimates are somewhat lower than the 30,000 pneumococcal deaths estimated by O'Brien et al. [4], using different methodology, different population figures and included few studies from China. We believe that the pneumonia burden using the proportionality that we obtained from our review is an underestimate of the true pneumococcal pneumonia burden because of the difficulties encountered in identifying the proportion of pneumonia due to *S. pneumoniae* microbiologically. Thus, we also used proportions obtained by O'Brien et al from vaccine efficacy trials. By applying these proportions, pneumococcal pneumonia cases and deaths increased by more than two-fold. Our results were based on a systematic review of mostly prospective, community or hospital based studies. When compared with the results obtained by Rudan, et al., using the Chinese Maternal and Child Mortality Surveillance (MCMS) system, we found that our estimates were somewhat lower. Assuming that 50% of all pneumonia deaths in 2000 were attributed to *S. pneumoniae*, our estimates for pneumococcal pneumonia deaths, using microbiologic and O'Brien's parameters were conservative.

There are inherent difficulties encountered in conducting studies assessing disease burden which likely underestimates the true burden in developing countries. First, disease is often underestimated when children do not reach the health facilities where surveillance is being conducted for case ascertainment. In our review, none of the studies that estimated the burden adjusted for these missed cases. Second, in assessing the number of deaths due to pneumococcal pneumonia, we did not adjust for *S. pneumoniae* being more likely associated with severe and fatal disease. Rudan, et al, estimated that there were 22 million episodes of pneumonia in Chinese children aged <5 years of age in 2000 and that 30–50% of radiological and fatal pneumonia were due to *S. pneumoniae* [26]. In Beijing, 55% of children aged 1 month to 5 years who died of community-acquired pneumonia had *S. pneumoniae* identified in their lung tissues using newer microbiologic techniques such as in situ PCR and/or Southern blotting [27]. Third, the hospital-based studies determining etiologies of pneumonia and meningitis which were included in this analysis were mostly conducted in tertiary hospitals, where patients were likely more severe and thus may not be representative of the spectrum of diseases in the population. Studies in Bangladesh demonstrated that compared with hospital-based designs, more *S. pneumoniae* cases can be identified through community-based studies [28], [29]. Fourth, the overuse of antibiotics in the early stage of illness could lower the culture positivity rate. In Asia, where antibiotics are readily available without prescriptions, a study conducted in China revealed high prior antibiotic usage among patients presenting in city/county hospitals, township hospitals and clinics at 96.6%, 88.0% and 78.6% respectively [30]. In a bacteremia surveillance in Thailand conducted in 2005–2006, it was estimated that prior antibiotic use reduced pneumococcal bacteremia incidence by 32% overall and 39% in children <5 years of age [31]. While other modalities such as antigen detection have been used in identifying pneumococcal infection and resulted in higher incidence rates [32], [33], culture remains to be the gold standard in detecting pneumococcal infections.

Certain limitations were specific to our study. First, we included studies with substantial differences in quality. Nonetheless, after stratifying by the quality of articles, no notable difference in parameters between those pooled from high quality studies and all studies in general (which could make the conclusion towards the opposite direction), was observed. Furthermore, precise numbers may not be as important as the patterns and trends emerging from the analysis. Second, we only included the three syndromes most commonly associated with *S. pneumoniae* and we have missed on other diseases associated with this disease, contributing further to the underestimation of the PD burden. Moreover, non-standardized definitions of pneumonia were used in some studies which may have further affected our estimates. Third, most of the studies combined reports from invasive and non-invasive isolates for serotyping and antimicrobial resistance, limiting our ability to predict serotype coverage and the potential for preventing antimicrobial resistance by PCVs against IPD. Lastly, we included data from studies conducted >20 years ago to calculate PD burden. This allowed inclusion of studies with substantial heterogeneity as laboratory procedures and diagnostic capabilities may have changed. Comparison of data thru time showed an increased occurrence of pneumonia in the 1990 s compared to the 1980 s, with no change in mortality. Moreover, the substantial economic development in China in the past 20 years may have lead to changes in sanitation, living conditions and access to health care. We applied the random effects model throughout the analysis to provide reasonable confidence intervals.

Twenty-seven percent of patients presenting with pneumonia or meningitis were colonized by *S. pneumoniae*. While nasopharyngeal isolation only indicates colonization and does not identify *S. pneumoniae* as the causative agent, a previous case-control study conducted in Beijing revealed that children with pneumonia were three times more likely to be colonized by *S. pneumoniae* in their nasopharynx compared to controls [34] and therefore may be helpful in identifying serotypes that are circulating.

Data regarding serotype distribution of *S. pneumoniae* isolates were limited to four studies that combined mixed invasive and non-invasive results. We were unable to disaggregate the serotype distribution of only invasive isolates, except in one study. Thus, the serotype coverage of the currently available pneumococcal conjugate vaccines was based on mixed invasive and non-invasive isolates. Results from sputum and hypopharyngeal aspirates may be contaminated from colonizing organisms and certain serotypes of *S. pneumoniae* have a propensity for colonization without necessarily causing disease. However, it is believed that invasive disease originates from colonization [35] and serotype distribution of colonization is an indicator of the organisms circulating in the community. It is important to note that three (19A, 19F and 14) of the most common serotypes causing invasive disease were also among the most common serotypes (19F, 19A, 23F, 6A, 6B and 14) seen from mixed specimens. This distribution was consistent with findings reported by Xue L, et al. and by Xiao SK, et al., both from 2010 which included data from different provinces in China [36], [37]. The circulating serotypes were comparable to those seen in other countries. Serotype 19A is also increasing in importance especially in countries that have been using PCVs in their national programs [38], [39]. When compared with the national survey conducted between 1982 and 1985, serotypes 1, 2 and 4 were less frequently isolated [40]. Based on our review, the currently available conjugate vaccines are expected to cover 79.%–88.4% of circulating serotypes in China. This is an encouraging finding for policy-makers to accelerate the introduction of pneumococcal vaccines in China.

Resistance to penicillin and other antibiotics has increased dramatically worldwide over the past decades due to inappropriate antibiotic usage [41]. Antibiotic resistance was commonly observed with beta-lactams and macrolides in Asia. The Asian Network for Surveillance of Resistant Pathogens (ANSORP) documented very high prevalence rates (>60%) of erythromycin resistance among clinical isolates of *S. pneumoniae* in Taiwan, Korea, Japan and Vietnam during 1996–1997 [42]. In our review, data on antimicrobial resistance were from mixed specimens as we were unable to disaggregate results from invasive isolates only. Nevertheless, a trend towards increasing resistance to penicillin and erythromycin was also seen in our study. Two changes in resistance trends had been recently reported. First, pneumococci resistant to both erythromycin and penicillin had emerged [43]. Currently, 15% to 30% of *S. pneumoniae* worldwide are multidrug-resistant (MDR) (i.e., resistant to ≥3 classes of antibiotics) [44], narrowing the clinical choice for treatment of PD. Second, only seven out of >90 serotypes (6A, 6B, 9V, 14, 19A, 19F, 23F) had been associated with the most resistant isolates [45]. Extensive usage of the 7-valent PCV has been shown to reduce antibiotic-resistant IPD in young children and older persons since five (6B, 9V, 14, 19F and 23F) of the resistant serotypes are contained in the 7-valent PCV. In China, where the 7-valent PCV is available in the private health setting only and uptake of the vaccine is low, it is interesting to note that serotype 19A is increasing in some areas [46]. This suggests that antimicrobial usage may be driving the circulation of certain antibiotic resistant serotypes in the community. While reducing antimicrobial pressure through stricter prescription policies has been initiated in China and will control circulation of antibiotic-resistant causing pneumococcus, use of the 19A-containing 13-valent PCV will likely substantially reduce PD in children.

The absence of population-based IPD surveillance studies in the past three decades in China has precluded us from providing more accurate point estimates of PD burden, however, based on our analysis *S. pneumoniae*-associated disease remains to be one of the leading causes of childhood deaths in China. With increasing antibiotic resistance and emergence of multidrug-resistant bacterial strains, introducing pneumococcal vaccines into the routine immunization program should be considered in China. Population-based studies are clearly warranted.
